# The P2Y_1_ receptor in the colonic myenteric plexus of rats and its correlation with opioid-induced constipation

**DOI:** 10.1186/s12876-024-03119-9

**Published:** 2024-01-08

**Authors:** Yuqiong Zhao, Huijuan Luo, Xiaojie Ren, Binghan Jia, Jinzhao Li, Lixin Wang, Junping Li

**Affiliations:** 1 Department of Human Anatomy and Histoembryology, College of Basic Medical Sciences, Ningxia Medical University, 1160 Shengli Street, 750001 Yinchuan, Ningxia Hui Autonomous Region P.R. China; 2 The Medical Laboratory Center of General Hospital of Ningxia Medical University, 804 Shengli Street, 750001 Yinchuan, Ningxia Hui Autonomous Region P.R. China

**Keywords:** µ opioids receptor (MOR), P2Y_1_ receptors, Opioid induced constipation (OIC), MRS2500, Colonic motility

## Abstract

This study was designed to explore the expression changes of P2Y_1_ receptors in the distal colonic myenteric layer of rats. An opioid induced constipation(OIC) rat model was generated by intraperitoneal (i.p) injection of loperamide. At 7 days post-treatment, the model rats were assessed by calculating the fecal water content and the gastrointestinal transit ratio. The immunofluorescence (IF)-based histochemical study was used to observe the distribution of P2Y_1_ receptors in the distal colonic myenteric plexus. Western blotting (WB) was performed to evaluate the expression changes of P2Y_1_ proteins in the myenteric layer, and the electrophysiological approaches were carried out to determine the regulatory roles of P2Y_1_ receptors on distal colonic motor function. IF showed that P2Y_1_ receptors are co-expressed MOR in the enteric nerve cells of the distal colonic myenteric plexus. Moreover, the WB revealed that the protein levels of P2Y_1_ were significantly decreased in the distal colonic myenteric layer of OIC rats. In vitro tension experiments exhibited that the P2Y_1_ receptor antagonist MRS2500 enhanced the spontaneous contraction amplitude, adding EM2 and β-FNA did not have any effect on MRS2500. Therefore, P2Y_1_ receptor expression could be associated with the occurrence of OIC in this rat model and the regulation of colonic motility by MOR may be related to the release of purine neurotransmitters such as ATP in the colonic nervous system.

**Supplementary Information:**

The online version contains supplementary material available at 10.1186/s12876-024-03119-9.

## Introduction

Opioids are widely recommended to treat various types of chronic and acute pain. Even though opioids are effective, their usage does come with adverse events [1]. One of the most adverse events is opioid-induced bowel dysfunction, and more specifically opioid-induced constipation (OIC), which can persist throughout the opioid treatment period. The pathogenesis of these adverse events is well known as the consequence of the action on µ-opioid receptors (MORs) in the peripheral nervous system [1, 2]. MORs are abundantly distributed on the neuronal cell surface in the enteric nervous system (ENS) along the gastrointestinal tract (GI). Studies have shown that opioids and their agonists mainly regulate the movement of gastrointestinal smooth muscles through the activation of MORs in the ENS [3]. So hyper-activation of MORs induces suppressed peristaltic contractions, resulting in constipation, which is the most common side-effect of therapeutically administered opioids [4, 5]. Furthermore, opioid-mediated MOR activation can inhibit the long-distance transport of intestinal contents along the colonic lumen, and increase the tension of smooth muscles, thereby inducing constipation. However, the specific molecular mechanism that underlies the constipating symptoms is not clearly understood to date [4, 6].

The GI motility is primarily modulated by the non-adrenergic non-cholinergic inhibitory neurons through the secretion of neurotransmitters, such as α purine, adenosine triphosphate (ATP), nitric oxide (NO), and carbon monoxide (CO), which stimulate cycles of hyperpolarization and relaxation of smooth muscles, thereby maintaining the normal function of gastrointestinal smooth muscle movement [7, 8]. In vitro studies have shown that electrical stimulation of inhibitory nerve cells releases inhibitory neurotransmitters that induce the inhibitory junction potential (IJP) of colonic circular muscle cells. Inhibitory neurotransmitter-induced IJP-mediated colonic smooth muscle relaxation can occur in two ways- a purinergic, large amplitude, fast IJP (fIJP), and a NO-dependent subdued, low amplitude slow IJP (sIJP) [9–11]. ATP transmitter serves as an essential component of purinergic signaling across the enteric musculomotor nerve terminals. In the GI, ATP regulates the functional activity of the colonic P2Y_1_ receptors by acting as one of the ligands [12]. The fIJP is highly sensitive to the P2Y_1_ receptor antagonist 2-iodo-N6-methyl-(N)-methanocarba-2’-deoxyadenosine-3’,5’-bisphosphate (MRS2500), while sIJP exhibits a higher sensitivity to the NO synthase inhibitor N^G^-nitro-L-arginine methyl ester (L-NAME) [10, 11].

P2Y_1_ belongs to the P2Y receptor superfamily of purinergic G-protein coupled receptors (GPCRs) which are stimulated by a variety of nucleotides. MRS2500 has demonstrated its high potential and selectivity in specifically blocking the fIJP signals but not the sIJP ones in colonic circular muscle cells [10, 13]. Previously we showed that the P2Y_1_ inhibitor MRS2179 aggravated the constipation symptoms in this OIC rat model, and the effect of ATP is similar to the MOR antagonists naloxone [14].

Therefore, we hypothesized that the colonic motility might be functionally regulated by the MOR-P2Y_1_ receptor cross-talk. Here, we determine the association between P2Y_1_ and OIC by examining the distribution of P2Y_1_ in the distal colonic myenteric plexus and examined the electrophysiological parameters of colonic smooth muscle cells to explore the underlying mechanism of P2Y_1_ receptor-mediated regulation of colonic motility following opioid administration in rats.

## Materials and methods

### Animals used

*Sprague-Dawley* (SD) male rats (6 weeks old; weighing 180-200 g; n = 44) were selected for this study. The grade of this batch of rats is SPF level, number: 10,752,309,202,200,156; the license number of the Animal Center of Ningxia Medical University: SCXK (Ning) 2020-0001. All protocols described below were approved by the Committee of the Ning Xia Medical University (Yin Chuan, China). Additionally, the research was reported consistent with the criteria provided by ARRIVE (https://arriveguidelines.org). Animals were acclimatized to the specific-pathogen-free environment 2–3 days before the start of the experiment with ad libitum access to food and water.

### Immunofluorescence (IF) assay

2 untreated rats were anesthetized by isoflurane inhalation and the colon was exposed along the midline of the abdomen. In our study, the distal colon segments were washed with 0.01 mol/l PBS solution and fixed with paraformaldehyde (PFA). Both ends were ligated and stored at 4℃ for 8 h. After 24 h in 30% sucrose solution, the preparations were cut along the mesentery margin and stored again in 30% sucrose solution. To prepare the whole mount for colonic tissues, we separated the mucosa, submucosa, and inner circular muscle layers from the outer longitudinal muscle layer, which was attached to the myenteric plexus. The tissues were then collected in the 0.01 mol/l PBS solution and subjected to duplex immunofluorescence (IF) staining for P2Y_1_ versus neuronal nuclei antigen (NeuN) or MOR, and ATP versus MOR. The tissue samples were incubated with primary antidodies as followes: i) anti-P2Y_1_ (rabbit polyclonal, NBP1-30741; NOVUS, Colorado, USA; 1:200) plus anti-NeuN (mouse monoclonal, Cat. No. 104,224; Abcam, Cambridge, UK; 1:200) antibodies; ii) anti-P2Y_1_ (rabbit polyclonal, NBP1-30741; NOVUS, Colorado, USA; 1:200) plus anti-MOR (Guinea pig polyclonal, Cat. No. NB100-1618; NOVUS, Colorado, USA; 1:200), and iii) anti-ATPB (mouse monoclonal; cat. no. 14,730; Abcam; 1:200) plus anti-MOR (Guinea pig polyclonal, Cat. No. NB100-1618; NOVUS, Colorado, USA; 1:200) antibodies in 0.01 M phosphate-buffered saline (PBS) containing 5% (v/v) fetal calf serum (Cat. No. 10,099,141 C; Gibco; Thermo Fisher Scientific, Inc.) and 0.3% (v/v) Triton X-100 (Solarbio; Cat. No. T8200) for 1 h at room temperature (RT), followed by 48 h at 4 °C. Next, tissue sections were rinsed with 0.01 M PBS 3 times and incubated with the corresponding pair of fluorescein-labeled secondary antibodies for three groups respectively: i) Alex488 labeled donkey anti-rabbit IgG (Cat. No. 6798; Abcam, Cambridge, UK; 1:500) and Alex594 labeled goat anti-mouse IgG (Cat. No. 150,116; Abcam, Cambridge, UK; 1:500); ii) Alex488 labeled donkey anti-rabbit IgG (Cat. No. 6798; Abcam, Cambridge, UK; 1:500) and Alex594 labeled goat anti-guinea pig IgG (Cat. No.150,188; Abcam, Cambridge, UK; 1:500); iii) Alex488 labeled donkey anti-mouse IgG (cat. no.6816; Abcam, Cambridge, UK; 1:500) and Alex594 labeled goat anti-guinea pig IgG (Cat. No.150,188; Abcam, Cambridge, UK; 1:500) antibodies for 2 h at RT, then washed 3 times with 0.01 M PBS. Finally, the slices were placed on the glass slides and covered with coverslips, along with a fluorescent encapsulated mounting medium (cat no. ab104139; Abcam). The specimens were imaged by a fluorescent microscope (40x magnification; SOLYMPUSBX51; Olympus Corporation).

### OIC Model

Rats were randomly divided into the OIC, normal saline group (NSG), and normal control group (NCG). For the OIC group, rats were given an intraperitoneal (i.p) injection of loperamide hydrochloride dissolved in 0.9% normal saline (4 mg/kg, 1 ml/100g) [15] twice a day for 7 days. For the NSG, 0.9% of normal saline (1 ml/100 g) was i.p injected twice a day for 7 days, while the rats in the NCG did not receive any treatment.

### Gastrointestinal motility

1. On the seventh day of modeling, the traits of rat feces were observed and recorded. Rat fecal samples were scored according to as follows: 1 = dispersed hard block; 2 = small sausage-like pieces; 3 = the presence of cracks on the sausage-like surface; 4 = the sausage-like surface was smooth and soft; 5 = soft lumps but clearly defined; 6 = pasty but unclear; 7 = watery feces [16]. 1–2 were considered constipation, 3–4 for normal fecal, and 5–7 for diarrhea.
2. Fecal water Content. Rat were housed individually without food and water for 1 h, and every fecal pellet was collected during this period. Fecal pellets were weighed as wet weight and weighed again as dry weight after placing in a 60 °C for 24 h. The fecal water content percentage was estimated by water content (in %) = 100 × (wet weight - dry weight) / wet weight.
3. NCG (n = 6), NSG (n = 6) and OIC (n = 6) rats were fasted for 12 h and 1mL of Indian ink (1 ml; Cat. No.16,060; biotopped) was orally administered; rats were randomly selected from each group and at 1.5 h post-treatment to measure the length of the intestine and the distance traveled by the ink.

Gastrointestinal transit ratio (%) = the distance of ink movement **/** the length of intestine × 100%.

### Western blotting (WB)

Rats were anesthetized by isoflurane inhalation and the colon was exposed along the midline of the abdomen. In our study, the tissue samples of the distal colonic muscle layer from NCG (n = 6), NSG (n = 6) and OIC (n = 6)rats were used for protein extraction using a commercial protein extraction kit (cat. no. KGP-2100; Jiangsu KeyGen Biotech). The protein concentration was determined by a bicinchoninic acid protein assay (cat. no. KGP-2100; Jiangsu KeyGen Biotech). Protein samples (3.6 µg/µl; 10 µl/ well) from each group was pipetted into a well of a 10% SDS-PAGE, cut the Polyvinylidene Fluoride (PVDF) membrane of appropriate size according to the molecular weight, soak it in methanol for 3 min, then assemble it in the order of rotating film clip black side, filter paper, gel, PVDF, filter paper, rotating film clip white side, and finally transfer on the membrane at 200 mA constant by the condition of 1.5 min/1KDa. The membrane was blocked by incubating in non-fat 5% skim milk for 2 h at room temperature before adding the following primary antibodies: anti-P2Y_1_ (rabbit polyclonal; cat. no. 85,896; Abcam, Cambridge, UK; 1:1000), anti-MOR (rabbit monoclonal; cat. no. ab134054; 1:1000; Abcam), anti-ATPB (mouse monoclonal, cat. no. 14,730; 1:1000; Abcam), and anti-GAPDH (mouse monoclonal, Cat. No. TA08; ZSGB-BIO, CHN; 1:1000) antibodies, then for 1 h at room temperature and allowed to incubate at 4 °C for overnight. After that, TBST was used to wash thrice to clear off any unbound primary antibodies, then the membrane was incubated with the respective secondary antibodies, either HRP-goat anti-mouse IgG (Cat. No. ZB-2305; ZSGB-BIO, CHN; 1:3000) or HRP-goat anti-rabbit IgG (Cat. No. ZB-2301; ZSGB-BIO, CHN; 1:3000) conjugated for 1 h at room temperature, and washed with TBST. The ECL reagent (cat. no. BMU101-CN; Abbkine) was added, and the images were captured by a chemical imaging system (Amersham Image 600). The gray value of each band was determined using ImageJ software and compared to the relative internal reference bands (target protein gray value/internal reference protein gray value).

### Mechanical test

#### Solutions and Drugs

The Krebs solution was prepared by dissolving the following components in the aqueous solution: 5.9 mM potassium chloride (KCl), 1.2mM sodium di-hydrogen phosphate (NaH_2_PO_4_), 1.2mM magnesium chloride (MgCl_2_), 120.6 mM sodium chloride (NaCl), 15.4mM sodium bicarbonate (NaHCO_3_), 2.5mM anhydrous calcium chloride (CaCl_2_), 1.5mM anhydrous glucose, MOR agonist EM2 (2µM; TOCRIS), MOR antagonist β-funaltrexamine hydrochloride (β-FNA; 10µM; TOCRIS), P2Y_1_ receptor agonist adenosine-5′-[β-thio]diphosphate trilithium salt (ADPβS; 10µM; Sigma), and P2Y_1_ receptor antagonist MRS2500 (1µM; TOCRIS).

The rats were anesthetized by isoflurane inhalation and the colon was exposed along the midline of the abdomen. The distal colon segments (below the splenic flexure of the colon) of rats (n = 6) were harvested by cutting it along the mesenteric border in a bathtub filled with Krebs solution. The distal colon segment was vertically hung in the direction of the circular muscle in the tension sensor. The tissue samples were equilibrated in 95% O_2_ + 5% CO_2_ and incubated under 1G at 37 °C for 1 h. When the distal colon segment showed stable contraction, the reagents were added according to the following order:

①EM2→β-FNA; β-FNA→EM2

After spontaneous contraction of the distal colon tissue segment occurs, EM2 is first added and observed for 10 min before continuing to be added β-FNA, record the experimental results. Reverse the order of adding reagents and wait for spontaneous contraction in the distal colon tissue segment before adding β-FNA firstly, observe for 10 min, then continue to add EM2 and record the experimental results.

②EM2→ADPβS; ADPβS→EM2

After spontaneous contraction of the distal colon tissue segment occurs, EM2 is first added and observed for 10 min before continuing to add ADPβS, recorded the experimental results. Reverse the order of reagent addition and add ADPβS first when spontaneous contraction occurs in the distal colon tissue segment, after observing for 10 min, continue to add EM2 and record the experimental results.

③MRS2500→EM2

After the spontaneous contraction of the distal colon tissue segment occurs, MRS2500 is first added and observed for 10 min, and then, EM2 is added and the experimental results are recorded;

④MRS2500→β-FNA

After the spontaneous contraction of the distal colon tissue segment occurs, MRS2500 is first added and observed for 10 min before continuing to be added β- FNA, record the experimental results.

The pharmacological experiments were conducted using a constant temperature in vitro smooth muscle system (HW-200 S; Chengdu Taimeng Software Co., Ltd.; China), and the experimental data was collected and analyzed using the BL420 biological signal acquisition and analysis system (TM-WAV software version 2.0; Chengdu Taimeng Software Co., Ltd.; China).

Tissues used for the IF, WB and Mechanical test experiments were obtained from the distal colonic area of the gut that could not be effectively repaired after being collected. Rats were euthanized via cervical dislocation while still under anesthesia.

### Statistical analysis

The GI transit ratio, fecal water content, WB and mechanical test were analyzed by one-way analysis of variance followed by Tukey’s post hoc test. SPSS 17.0 (SPSS, Inc.) and GraphPad 8.3.0 statistical software (GraphPad Software, Inc.) were used to perform all statistical analyses. *P*-value of < 0.05 was considered statistically significant.

## Results

### P2Y_1_ receptors co-localize with NeuN on the enteric neuronal surfaces in the rat colonic myenteric plexus

**Fig. 1:**
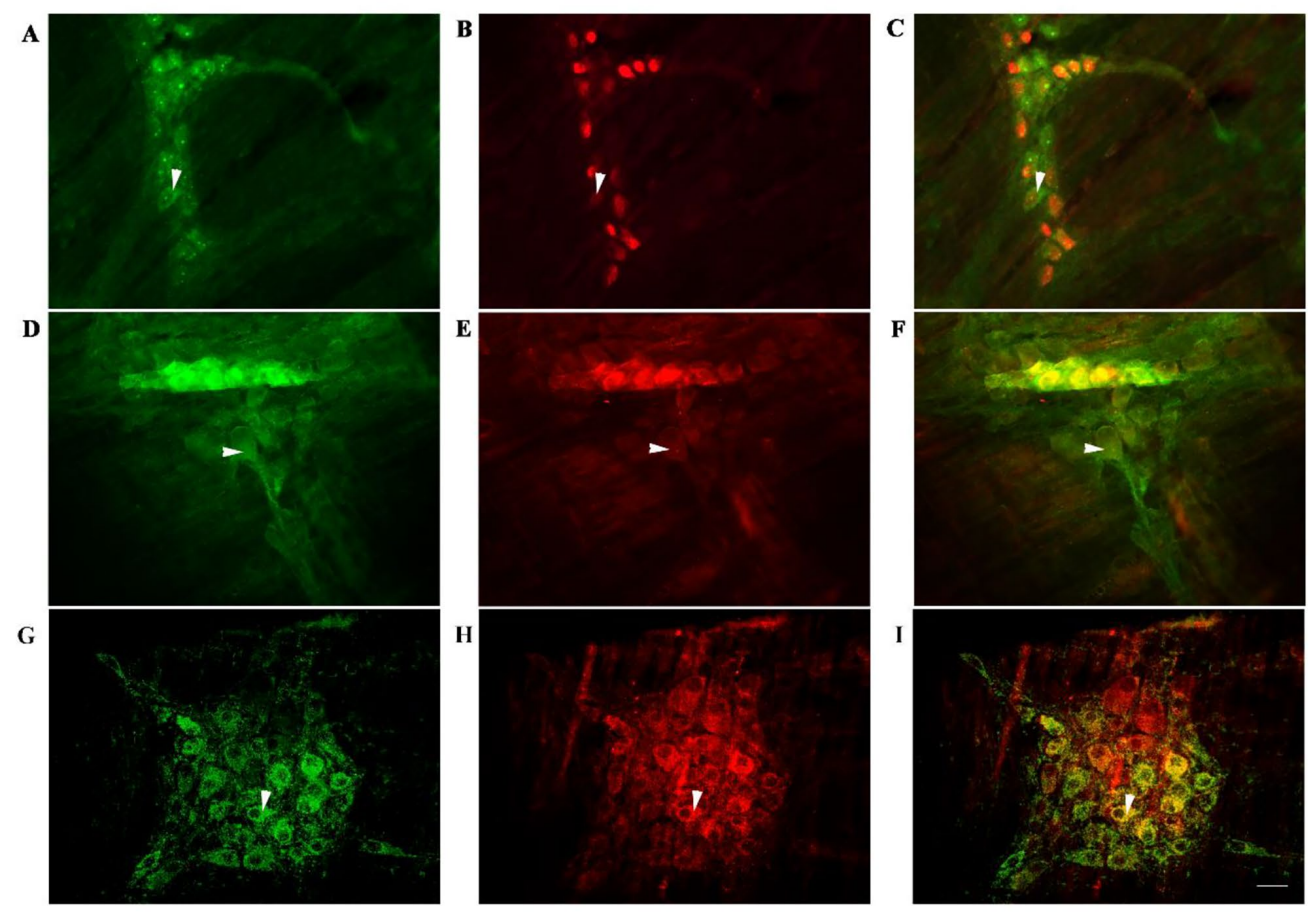
The immunofluorescence (IF) histochemistry indicates the co-existence of various opioid response factors in the myenteric plexus of a normal rat colon. (**A**) Representative images showing the P2Y_1_ immunoreactivity; (**B**) the NeuN staining on the same section; (**C**) the co-localization of P2Y_1_ and NeuN on the enteric neurons. (**D**) Representative IF images showing the P2Y_1_ staining; (**E**) the MOR immunoreactivity; (**F**) IF image showing the co-localization of P2Y_1_ with MOR. (**G**) Representative IF images showing the ATP staining; (**H**) the MOR immunoreactivity; (**I**) IF image showing the co-localization of ATP with MOR. Scale bars, 20 μm

The IF-based histochemical analyses revealed that P2Y_1_-positive nerve cells were aggregated in the colonic myenteric plexus to form ganglia (Fig. 1). In the ganglia, a large number of P2Y_1_-positive nerve fibers can be seen passing through the cells (Fig. 1A). The P2Y_1_-positive nerve cell bodies were round or elliptical in shapes, and the P2Y_1_ expression was mainly located in the cell bodies and processes, emitting a long protrusion from the cell body (Fig. 1A and D). Moreover, these P2Y_1_-positive cells exhibited co-localization with NeuN marker in the intestine, indicating that these cells were enteric neurons (Fig. 1A-C). Furthermore, MORs were co-expressed with P2Y_1_ on the intestinal nerve cells, and a large number of MOR-positive nerve cell bodies were fibers surrounded the cell body of P2Y_1_-positive cells (Fig. 1F). In the ganglion, MOR and ATPBpositive markers were coexpressed in the intestinal nerve cells and the cell bodies of ATPB-positive nerve cell bodies were mostly elliptical in shape, and their positive markers are mainly located in the cytoplasm (Fig. 1G-I).

**Fig. 2:**
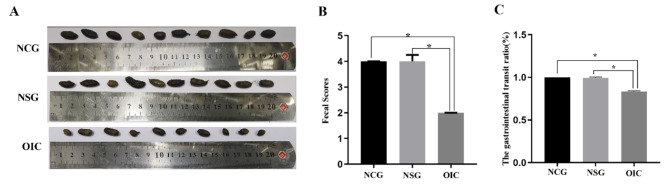
The fecal traits (**A**) and fecal scores (**B**) on the seventh day and gastrointestinal transit ratio (**C**). *P < 0.05. OIC, opioidinduced constipation; NSG, normal saline group; NCG, normal control group

The fecal of OIC rats became smaller and harder on the 7th day of modeling, which was close to the small sausage-like pieces with a score of 2, while the fecal morphologies of NCG and NSG rats were very similar, with a sausage-like appearance but a smooth and soft surface with a score of 4**(**Fig. 2A and B**)**. The GI transit ratio and fecal water content of the OIC rats were significantly lower than NCG and NSG animals on the seventh day of modeling **(**Fig. 2C and Table 1).

**Table 1:** I The fecal water content (%)

Group	NCG	NSG	OIC
Water content(%)	58.3 ± 3.1	55.6 ± 4.9	49.2 ± 1.6^ab^

The fecal water content was decreased in OIC (^a^*P* < 0.05, OIC vs. NCG; ^b^*P* < 0.05, OIC vs. NSG)

**Fig. 3:**
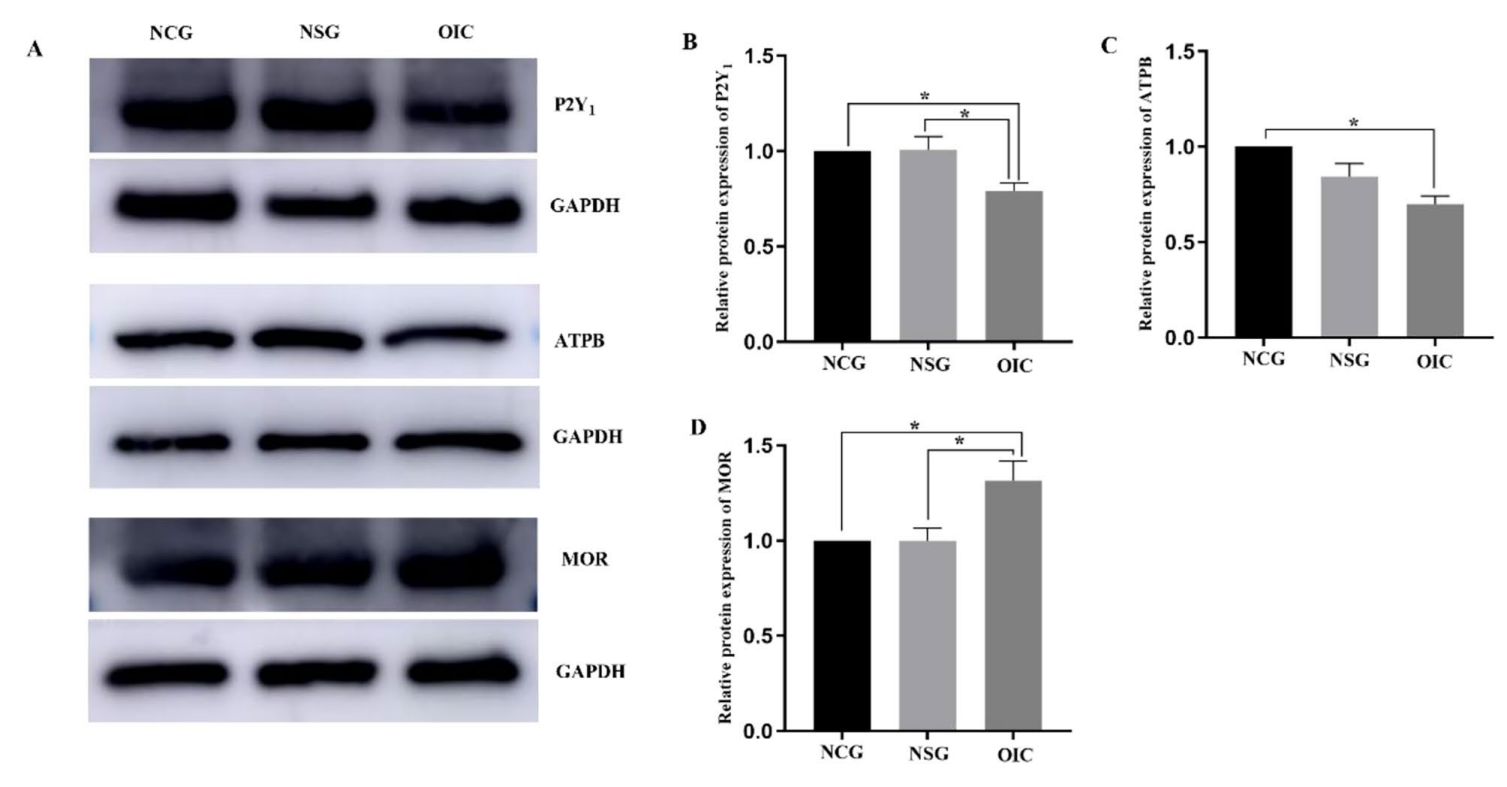
(**A-D**) Western bloting analysis showed the relative protein expression of P2Y_1_, ATPB and MOR in rat colonic muscle layer. *P < 0.05. OIC, opioid-induced constipation; NSG, normal saline group; NCG, normal control group; P2Y_1_, P2Y purinergic receptor 1; ATPB, ATP synthase subunit β

The protein levels of P2Y_1_ decreased in OIC rats. Studies have shown that ATP acts through Gq-coupled P2Y_1_ receptors, and then reduces the contractility of smooth muscle [12]. In this study, WB analysis revealed that MOR protein levels in the myenteric layer of OIC rats were significantly increased, while the P2Y_1_ and ATPB proteins were significantly decreased compared with those of the NCG and NSG rats (Fig. 3).

### Differential mechanical responses of enteric neurons to MOR and P2Y_1_ modulators

**Fig. 4:**
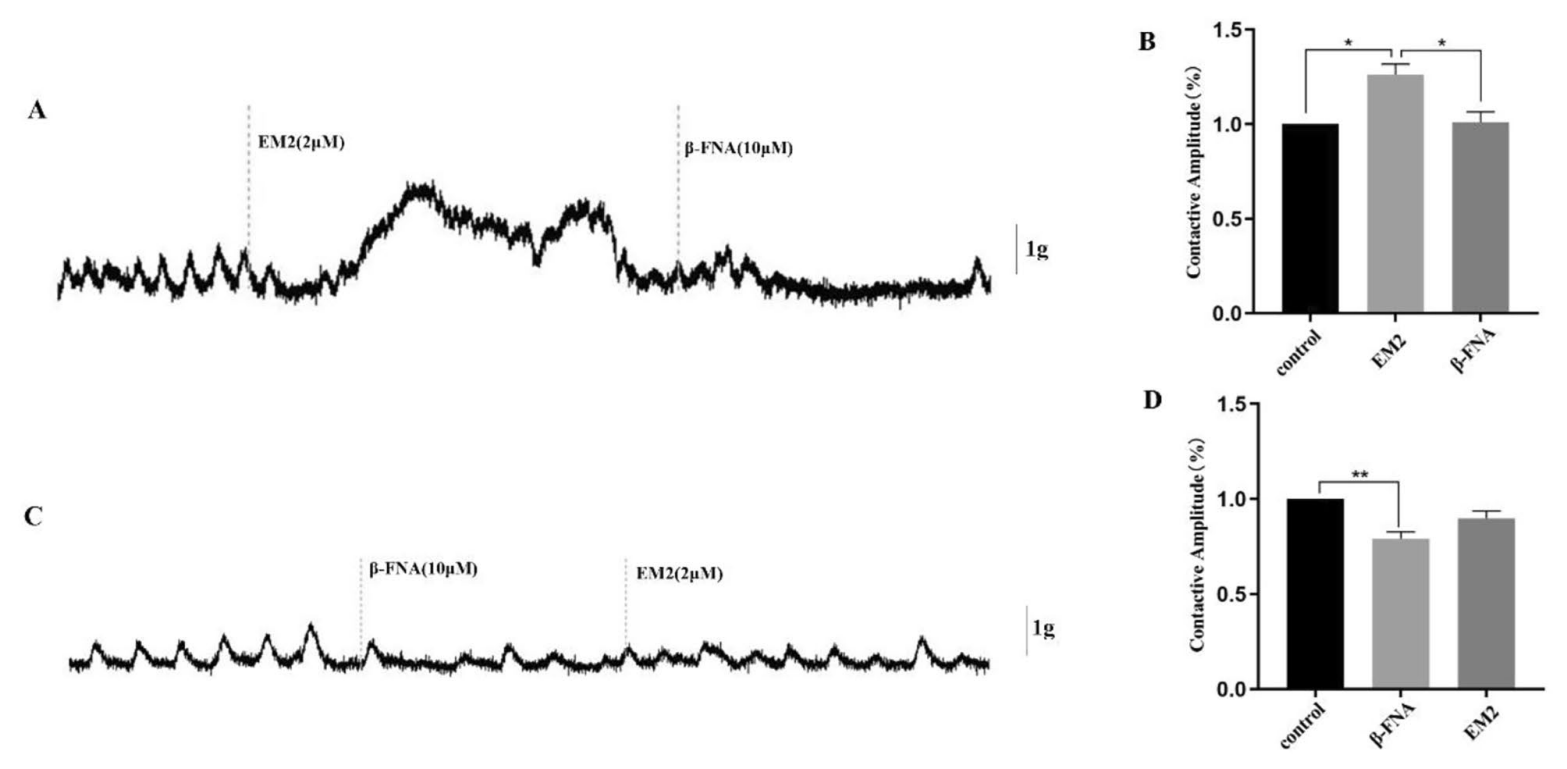
Spontaneous contractive amplide and effects of MOR antagonists (EM2 and β-FNA) on the motility of circular muscle strips from the rat colon. (**A-B**) EM2 increased the amplitude, while β-FNA significantly suppressed EM2-evoked contractions (*P<0.05). (**C-D**) The β-FNA decreased the amplitude of spontaneous contractions(*P<0.05). Conversely, EM2 had no obvious effect on the inhibition of β-FNA (*P>0.05)

**Fig. 5:**
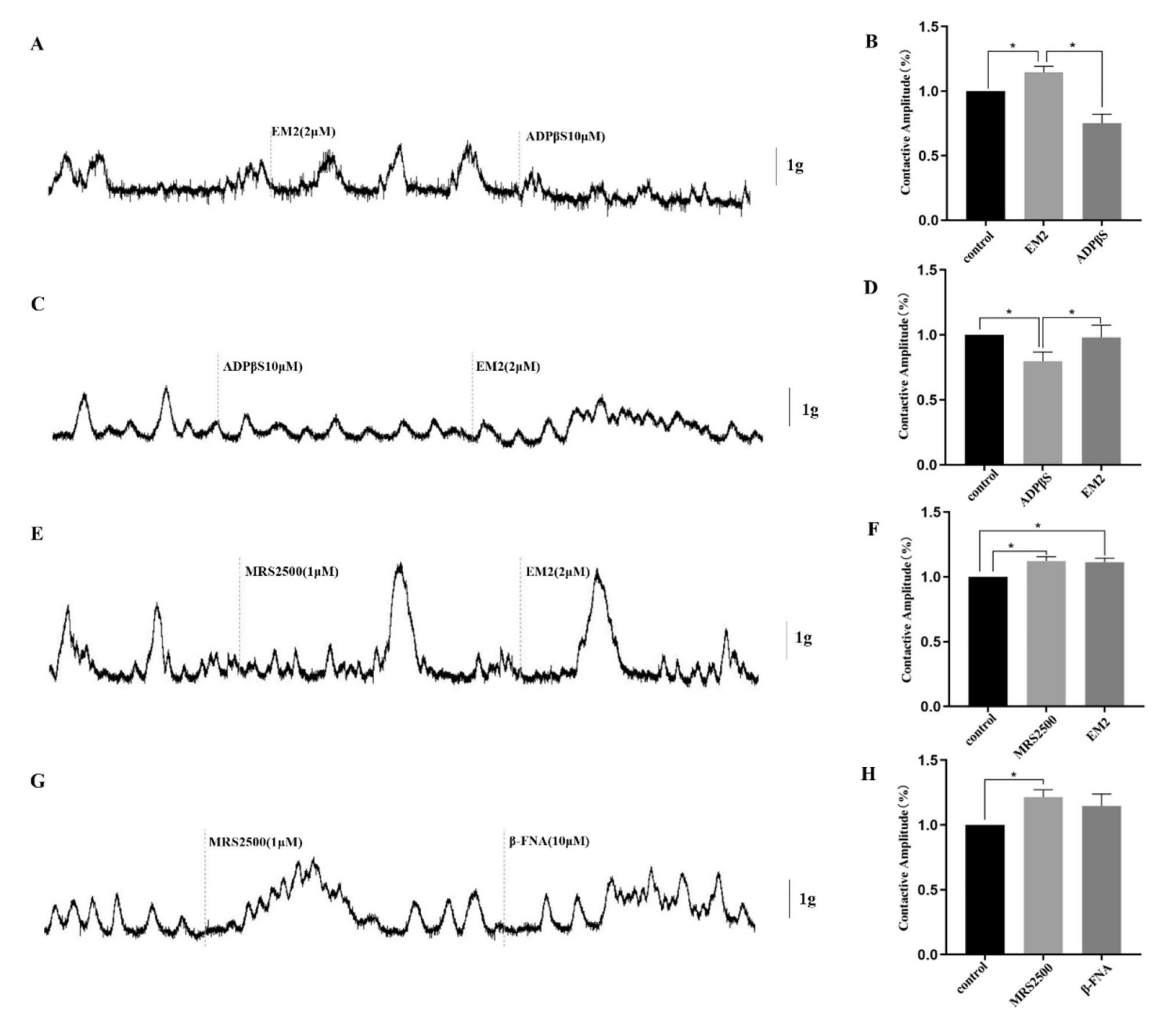
Spontaneous contractive amplide and the interaction among EM2, ADPβS (an agonist of P2Y_1_), and MRS2500 (an antagonist of P2Y_1_) on the motility of circular muscle strips of the rat colon. (**A-B**) ADPβS reduced the effect of EM2(*P<0.05). (**C-D**) ADPβS inhibited the spontaneous contraction, and the continued addition of EM2 alleviated this effect (*P<0.05). (**E-H**) MRS2500 promoted the distal colon spontaneous contractive amplide (*P<0.05), adding EM2 and β-FNA did not have any effect of MRS2500(*P>0.05)

In vitro, tension experiments showed that 1G of tension to the distal colon segment could induce regular spontaneous contractions, and then administration of the endogenous agonist EM2 of MOR significantly increased the spontaneous contractive amplitude of the distal colon segment (Fig. 4A and B). While the selective antagonist of MOR, β-FNA, significantly inhibited the effect of EM2 by reducing the spontaneous contractive amplitude of distal colon segments (Fig. 4A and B). Besides, when the order of addition of these two drugs was reversed, the spontaneous contractive amplitude of distal colon segments was significantly inhibited by β-FNA and could not be altered by EM2 exposure (Fig. 4C and D). Interestingly, ADPβS had a significant inhibitory effect on the spontaneous contractive amplitude of the distal colon segment, which was significantly abrogated by EM2 (Fig. 5C and D). Reversing the order of drug additions revealed that ADPβS significantly attenuated the amplitude of EM2-induced spontaneous contractions of distal colon segments (Fig. 5A and B). Moreover, the addition of the P2Y_1_ receptor antagonist MRS2500 significantly enhanced the amplitude of spontaneous contractions of distal colon segments, but continuely add EM2 had no significant modulatory effect on the MRS2500 activity (Fig. 5E and F). At the same time, when added in the reverse order, β-FNA also had no effect on MRS2500 on the spontaneous contraction of distal colon segments (Fig. 5G and H).

## Discussion

OIC is characterized by bowel frequency reduction, worsening or development of straining, a sensation of incomplete defecation, and harder fecal consistency [2]. Loperamide is an opioid receptor agonist that works by activating the MORs located in the myenteric plexus of the ENS. Upon binding to the MOR, loperamide decreases the activity of the myenteric plexus, which subsequently reduces the tone of the circular and longitudinal smooth muscles of the gut wall. This in turn reduces propulsion and extends the total stay time of luminal contents [15]. In this experiment, after 7 days of modeling, the fecal samples of OIC rats were observed to become smaller and harder, while the GI transit ratio and fecal water content of these OIC rats were significantly decreased. This was consistent with the characteristics of the OIC fecals in previous reports [2], indicating that the OIC rat model was successfully established in this study.

The ENS, also known as the gut-brain, is a neural network system composed of enteric nerve cells and glial cells in the GI tract. The ENS has a complete reflex pathway and can independently regulate the motor function of the GI tract even in vitro, including the submucosal plexus located in the submucosa and the myenteric plexus intestinal tract located between the longitudinal and circular muscles [17]. According to the morphological and electrophysiological properties, enteric neurons can be divided into primary afferent neurons, intermediate neurons, and motor neurons. Primary afferent nerve cells are mostly located in the submucosal plexus of the GI tract, and transmit various mechanical and chemical stimuli to the intermediate and motor nerve cell bodies in the enteric muscular plexus, thereby regulating the movement of GI smooth muscles [18]. Nerve cells that control and regulate the smooth muscle movement in the GI include both excitatory and inhibitory motor neurons. The inhibitory motor nerve cells are mainly non-cholinergic and non-adrenergic by nature, which mainly secretes crucial inhibitory neurotransmitters such as ATP to control GI motility [19]. MOR belongs to the GPCR family and is widely distributed across the nervous system. MORs function as the primary action sites for morphine and other opioids in the peripheral nervous system [7, 20].

In the GI, MOR is abundantly distributed in the ENS. Compared with other opioid receptors, MORs exhibit differential population and regional properties. It is more densely distributed in the colon than in the stomach and small intestine [7, 17, 20, 21]. Studies have shown that MOR mainly regulates the movement of GI smooth muscle by modulating the release of excitatory and/or inhibitory neurotransmitters, including ATP, CO, and NO [6, 7]. Notably, MOR usually inhibits the release of inhibitory neurotransmitters, like ATP, from enteric neurons [22]. ATP is a kind of purine transmitter, widely distributed in colonic nerve cells, and is a high-affinity ligand of the P2Y_1_ receptor. IF histochemical results revealed that there were P2Y_1_-positive enteric neurons in the myenteric plexus along with MOR and P2Y_1_ or ATPB co-expressing neurons in the rat distal colon of rats. This indicates that in the colonic nervous system, MOR and P2Y_1_ receptors have a morphological basis for forming a complete neuromodulation pathway. P2Y_1_ is a member of the purine receptor (GPCR) family. In the GI, ATP regulates the functional activity of the colon movement by activating certain receptors like P2Y_1_ [11, 23]. Here, the protein expression levels of P2Y_1_ receptor and ATPB were significantly decreased in the distal myenteric layers of OIC rats, while the levels of MOR were significantly increased. Our previous study has also shown that the P2Y_1_ receptor agonist ATP can significantly relieve the constipation symptoms in rats, while the P2Y_1_ receptor inhibitor MRS2179 aggravates these symptoms [14]. Overall, it is speculated that P2Y_1_ expression change may be associated with the occurrence of OIC, and expressions of MOR and P2Y_1_ were well-correlated with the OIC development in rats.

In vitro, mechanical stimuli such as tension-stretching can activate the ENS, triggering enteric nerve cells to release neurotransmitters that regulate the voluntary contraction of GI smooth muscles [9, 24–26]. The results of in vitro tension experiments showed that 1G of tension in the distal colon segment in the direction of the circular smooth muscles promoted spontaneous rhythmic contractions. Application of the high-affinity endogenous agonist EM2 of MOR further provoked the spontaneous contraction of distal colon segments, which was completely inhibited by a selective antagonist of MOR, β-FNA. Reversing the order of addition of these two drugs indicated that EM2 did not affect β-FNA in modulating the amplitude of spontaneous contractions of colon segments. It is reported that EM2 has a similar distribution pattern to MOR in vivo, especially in colon tissue [27]. This suggests that EM2 and β-FNA operate through MOR in the colonic nervous system, and regulate the movement of the colonic annular muscles, as well.

Importantly, the spontaneous contraction of colonic annular muscles is regulated by non-cholinergic and non-adrenergic neurotransmitters (ATP, NO, CO) and other inhibitory neurotransmitters [9]. The tension test demonstrated that the P2Y_1_ receptor agonist ADPβS inhibited the spontaneous contractive amplitude of the distal colon segment, and then the addition of EM2 blocked the effect of ADPβS. Reversing the order of drug additions revealed that ADPβS and EM2 had a reciprocal effect on the distal colonic muscle movement. In the GI, inhibitory neurotransmitters mostly include purines and nitro compounds, like ATP and NO [8]. Importantly, purine neurotransmitters play a leading role in the transmission of inhibitory neural information. They cause rapid hypertrophy of GI smooth muscle cells by activating P2Y_1_ receptors [28–31]. It is hypothesized that the addition of EM2 may inhibit the release of inhibitory neurotransmitters in colonic nerve cells, thereby promoting the spontaneous contraction of the colon, then ADPβS can activate the P2Y_1_ receptors on the surface of colonic smooth muscle cells to induce smooth muscle relaxation and inhibit the function of EM2. This notion further indicates that the inhibitory neurotransmitters released by EM2 blocking may be endogenous ligands of P2Y_1_ receptors. Studies have delineated that the P2Y_1_ receptor antagonist MRS2500 can completely block the effects of ADPβS, ATP, and other purine transmitters in the GI tract [32]. In this context, the results of the tension test highlighted that MRS2500 enhanced the spontaneous contractive amplitude of distal colon segments, and this effect could not be prevented by EM2 exposure. Moreover, the addition of β-FNA first had no impact on MRS2500 activity in controlling the spontaneous contractive amplitude of colon segments. The P2Y_1_ receptor is the dominant receptor among purinoceptors that regulates colonic motility [9]. In the colon, activation of P2Y_1_ receptors is an important pathway for neuro-mediated purinergic inhibitory responses [33]. It is suggested that the regulation of smooth muscle movement by EM2 after activating MOR on the surface of enteric nerve cells is mostly controlled by the release of purine neurotransmitters in colonic nerve cells that regulate P2Y_1_ receptor-mediated voluntary contraction of colonic annular muscles. Undoubtedly, MOR may also have regulatory effects on other inhibitory neurotransmitters in the colon.

To sum up, the results of the present study suggest that P2Y_1_ receptor expression could be associated with the occurrence of OIC in this rat model. Besides, the occurrence of OIC may be associated with MOR dysfunction leading to abnormal P2Y_1_ receptor function. Moreover, combined with the results of this experiment, it can be concluded that the regulation in the spontaneous contraction of colonic circular muscles by MOR may be related to the degree of release of purine neurotransmitters in the ENS, thereby regulating the function of P2Y_1_ receptors.

### Electronic supplementary material

Below is the link to the electronic supplementary material.

Supplementary Material 1

## Data Availability

The datasets used and/or analyzed during the current study are available from the corresponding author on reasonable request.

## Abbreviations

P2Y_1_: P2Y purinergic receptor 1
MOR: µ-opioid receptor
OIC: Opioid-induced constipation
EJP: Excitatory neuromuscular junction potential
IJP: Inhibitory neuromuscular junction potential
fIJP: Fast inhibitory junction potential
sIJP: Slow-acting neuromuscular junction potential
IF: Immunofluorescence
EM2: Endomorphin-2
ADPβS: Adenosine 5′-[β-thio] diphosphate trilithium salt
MRS2500: 2-iodo-N6-methyl-(N)-methanocarba-2’-deoxyadenosine-3’,5’-bisphosphate
β-FNA: Beta-Funaltrexamine
ENS: Enteric nervous system
AEs: Adverse events
GI: Gastrointestinal tract
