# Supplementary figures and images for: The P2Y1 receptor in the colonic myenteric plexus of rats and its correlation with opioid-induced constipation
Source: BMC Gastroenterol. 2024 Jan 8;24:23. doi: 10.1186/s12876-024-03119-9 (PMC10773096; doi:10.1186/s12876-024-03119-9)

original gels and blots of Fig3A

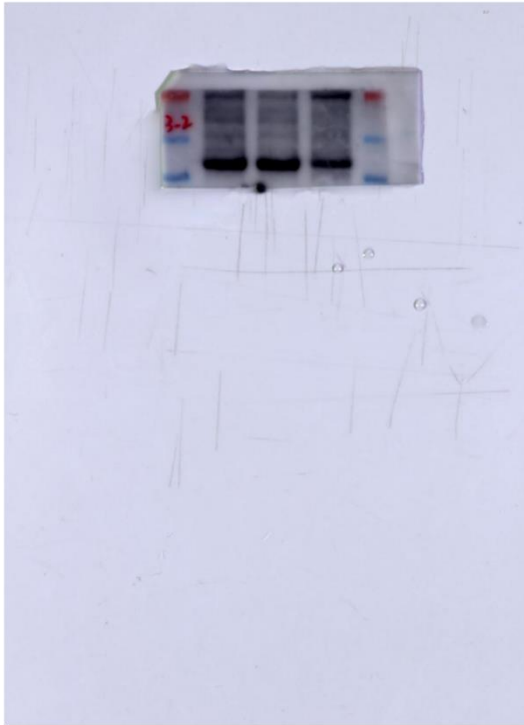

**P2Y1-3-2**

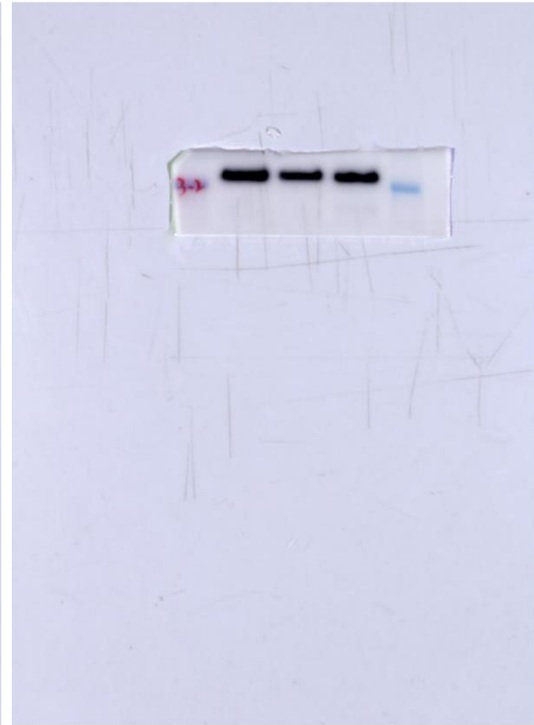

**GAPDH-3-2**

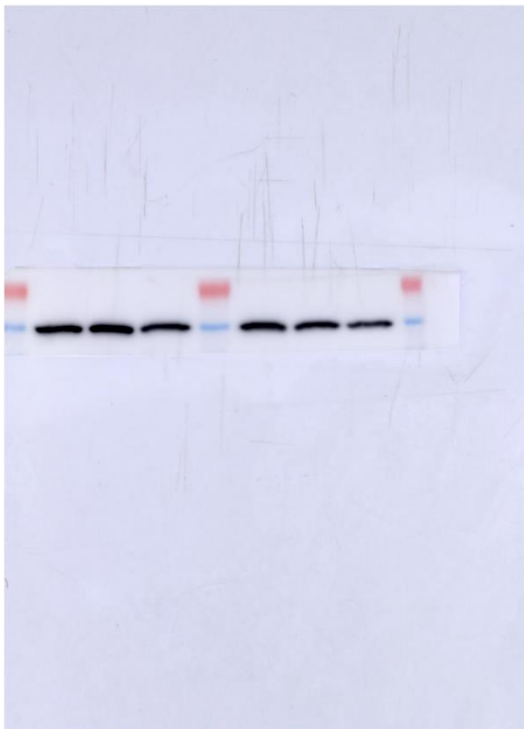

**ATP-2**

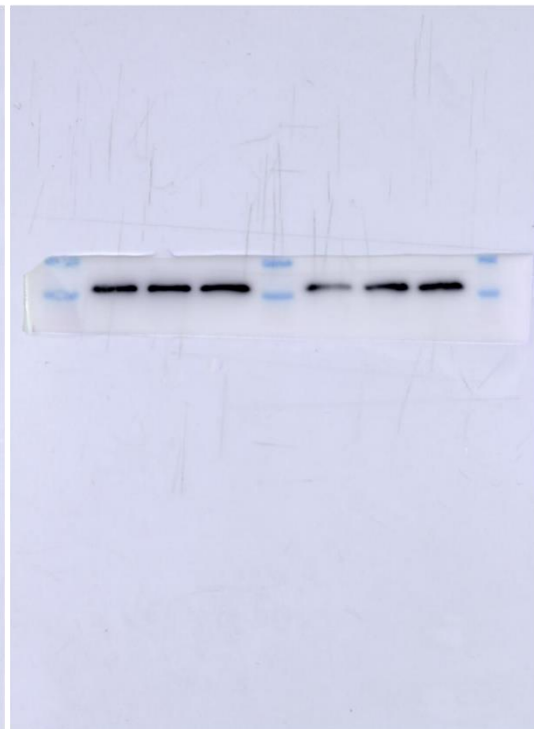

**GAPDH-2**

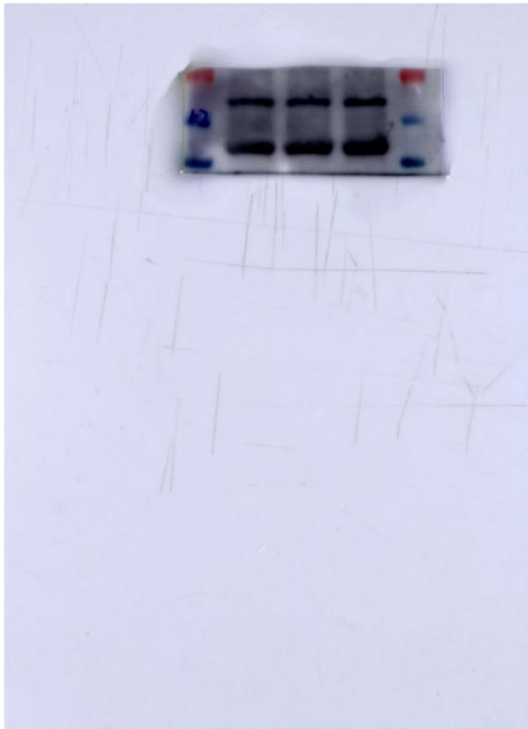

**MOR-1-2**

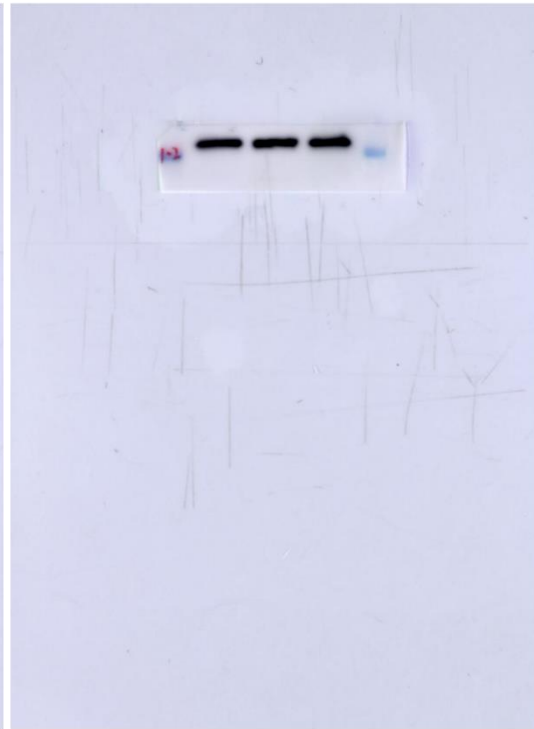

**GAPDH-1-2**

Supplement: Supplementary file 1 — Supplementary Material 1 [file 12876_2024_3119_MOESM1_ESM.pdf]
